# RNA Drug Delivery Using Biogenic Nanovehicles for Cancer Therapy

**DOI:** 10.3389/fphar.2021.734443

**Published:** 2021-12-24

**Authors:** Nuannuan Li, Yiying Sun, Yuanlei Fu, Kaoxiang Sun

**Affiliations:** ^1^ Key Laboratory of Molecular Pharmacology and Drug Evaluation (Yantai University), School of Pharmacy, Collaborative Innovation Center of Advanced Drug Delivery System and Biotech Drugs in Universities of Shandong, Ministry of Education, Yantai University, Yantai, China; ^2^ Shandong International Biotechnonlogy Park Development Co. Ltd, Yantai, China; ^3^ Key Laboratory of Nanomedicine and Advanced Preparations, Yantai Institute of Materia Medica, China, Yantai, China

**Keywords:** RNA delivery, cancer, biogenic materials, oligonucleotide, exosomes, cell membranes

## Abstract

RNA-based therapies have been promising method for treating all kinds of diseases, and four siRNA-based drugs and two mRNA-based drugs have been approved and are on the market now. However, none of them is applied for cancer treatment. This is not only because of the complexity of the tumor microenvironment, but also due to the intrinsic obstacles of RNAs. Until now, all kinds of strategies have been developed to improve the performance of RNAs for cancer therapy, especially the nanoparticle-based ones using biogenic materials. They are much more compatible with less toxicity compared to the ones using synthetic polymers, and the most widely studied biogenic materials are oligonucleotides, exosomes, and cell membranes. Particular characteristics make them show different capacities in internalization and endosomal escape as well as specific targeting. In this paper, we systematically summarize the RNA-based nano-delivery systems using biogenic materials for cancer therapy, and we believe this review will provide a valuable reference for researchers involved in the field of biogenic delivery and RNA-based therapies for cancer treatment.

## Introduction

RNA therapy, including mRNA and RNA interference (RNAi) therapeutics, has received substantial attention in this decade, especially with the emergence of the coronavirus disease 2019 (COVID-19) outbreak. This may be attributing their superiorities over other macromolecular drugs such as protein- and DNA-based treatments. Compared with protein-based therapeutics, RNA therapeutics are more cost-effective, and they can be manufactured rapidly on a large scale. In the meantime, these treatments have been demonstrated to display greater therapeutic efficiency than DNA drugs because they can act in the cytoplasm, whereas DNA therapeutics require entry into the nucleus (Wallis et al., 2019). mRNA-based therapies act by upregulating the expression of targeted proteins, and the successful outcomes of mRNA vaccines including BNT162b2 (Pfizer-BioNTech) and mRNA-1273 (Moderna) for COVID-19 prevention have brought RNA therapeutics to new heights (Khurana et al., 2021). On the contrary, RNAi therapies including microRNA (miRNA) and small interfering RNA (siRNA) act by complexing with RNA-induced silencing complex (RISC) in the cytoplasm to induce cleavage of the mRNA sequence and thus downregulate the expression of checkpoint proteins for disease treatment (Thody et al., 2020). The main difference between siRNAs and miRNAs is that siRNAs can degrade or inhibit mRNA translation with 100% complementarity, whereas miRNAs usually bind mRNA with incomplete complementarity (Khan et al., 2021). Consequently, siRNAs exhibit precise target specificity, and thus, they have been widely studied, including four approved siRNA drugs developed by Alnylam Pharmaceuticals: ONPATTRO^®^ (patisiran, 2018), GIVLAARI™ (givosiran, 2019), Oxlumo^®^ (lumasiran, 2020), and Leqvio^®^ (inclisiran, 2020) (Mikami et al., 2020). All these approved RNA drugs provide a strong rationale for exploring other RNA moieties as novel therapeutics for various diseases, particularly for diseases caused by gene dysregulation such as cancer.

**FIGURE 1:**
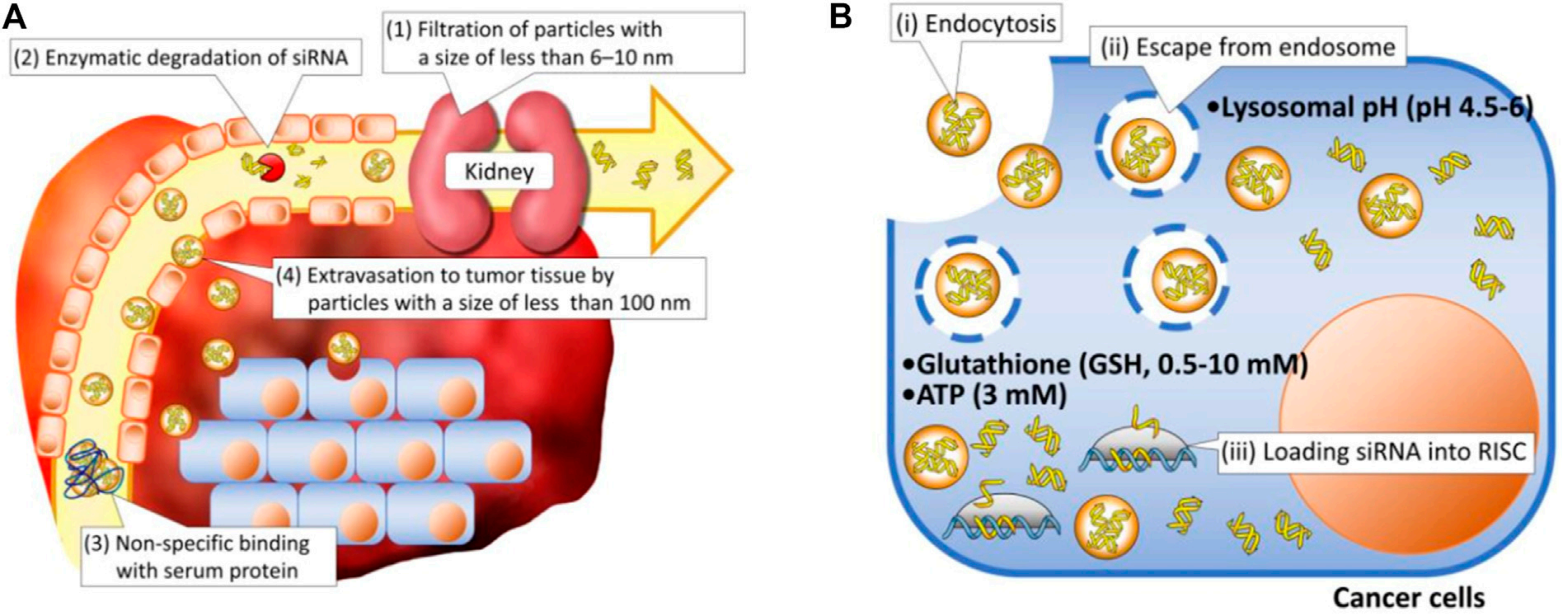
Schematic illustration of delivery barriers in **(A)** extracellular and **(B)** intracellular regions (Kim HJ. et al., 2016).

Although RNA drugs designed for cancer treatment have been examined in clinical trials for several decades, none has been successfully approved for clinical application. This is attributable to the complicated pathophysiological environments of cancers including the dense tumor stroma, collapsed blood vessels, immunosuppression, multidrug resistance, and hypoxia, which can promote tumor progression and prevent therapeutics from entering tumor tissue (Heinrich et al., 2021; Liu J. et al., 2021), as well as the intrinsic obstacles of RNAs such as their high molecular weight, high hydrophilicity, negative charge, and instability (Singh et al., 2020). Being ideal therapeutic agents, RNAs must overcome both extracellular and intracellular barriers. First, when injected directly into the bloodstream, naked RNA molecules must avoid enzymatic degradation, rapid renal filtration, and phagocytic entrapment because of their small size, poor stability, and immunogenicity (Kulkarni et al., 2019; Yarian et al., 2019). Second, once they reach tumor sites, the RNA molecules must overcome their intrinsic flaws regarding physicochemical properties such as a high molecular weight, negative charge, and high hydrophilicity, permitting them to efficiently cross negatively charged biological membranes with lipid bilayers and internalize into cancer cells. Finally, even after internalization into cells, few RNA molecules can escape endosomal entrapment, possibly because most extraneous agents are internalized through an endocytic and endo-lysosomal pathway. Lysosome, with their acidic environment and specific enzymes, can induce significant degradation of RNAs. All aforementioned barriers in extracellular and intracellular regions lead to the low therapeutic efficiency of RNAs (Figure 1) (Kim MS. et al., 2016).

To date, multiple chemical modifications have been reported to improve the stability and reduce the immunogenicity of RNA therapies (Khan et al., 2021). In addition, these strategies have been successfully implemented in clinical practice with the launches of givosiran, lumasiran, and inclisiran, which are constructed by conjugating siRNA to N-acetylgalactosamine molecules. However, endosomal escape remains an issue (Dammes and Peer, 2020). A better modality with endosomal escape ability is the lipid nanoparticle (LNP), which has been widely applied in the other approved RNA drugs. This is mainly because of the addition of ionizable lipids that can remain neutral under physiological conditions (pH 7.4) during systemic circulation but acquire positive charges at acidic pH for endosomal membrane destabilization (Schlich et al., 2021). However, the main obstacle is the delivery of RNA molecules to tumor sites other than the liver. The off-target effects, which can induce serious side effects and reduce the therapeutic efficacy, may result from the non-specific delivery and inability in immune escape of LNP. Thus, an ideal RNA delivery system for cancer treatment is the one which not only can protect RNAs from being destructed and have the capability for endosomal escape to release the RNAs into cytosol, but also possess high targeting ability to make the RNAs delivered into tumor sites and escape from the surveillance of immune systems.

**FIGURE 2:**
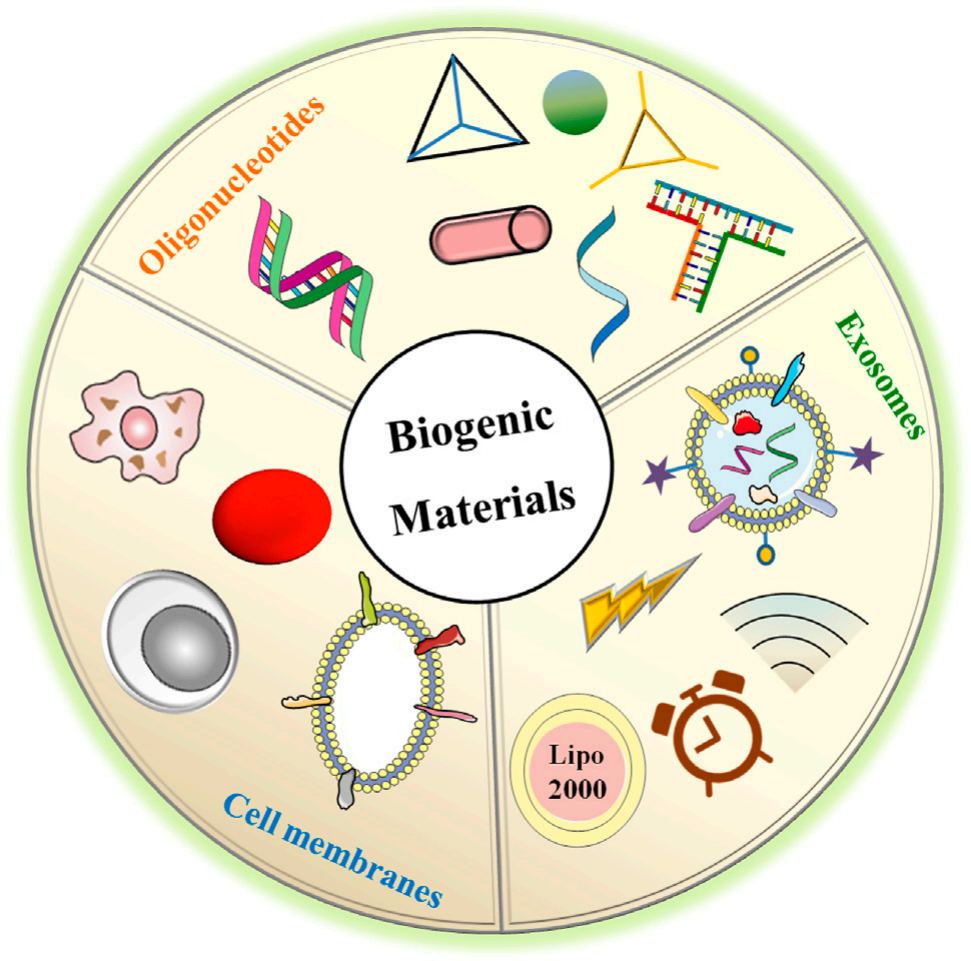
The biogenic materials used for RNAs delivery including oligonucleotides assemblies with different shapes, exosomes loading RNAs using various manners, and membranes derived from different cell types with specific characteristics.

Based on the aforementioned theories, biogenic nanovehicles such as oligonucleotides, exosomes, and cell membranes have been developed (Figure 2). They not only can protect RNAs from rapid renal filtration and destruction by enzymes, but also can exhibit high biocompatibility and low immunogenicity, permitting them to reduce organ toxicity encountered in cationic vectors (Lv et al., 2006) and protect RNAs from clearance by the immune system (Xu et al., 2021). Especially, exosomes and cell membranes exhibit intrinsic targeting ability via their surface receptors with recipient cell. In addition, exosomes have also been reported with high internalization efficacy and endosomal escape capacity *via* membrane fusion mechanism. Although oligonucleotide assemblies seem to show lower targeting ability than exosomes and cell membranes, the high programmability makes them with precisely controlled structures and functional sites for ligand attachments to induce enhanced tumor accumulation, cellular internalization, and endosome escape (Kim et al., 2019). Of course, these biogenic nanovehicles can also cooperate with other functional materials such as the cationic molecules polyethyleneimine (PEI) with efficient endosomal escape (Jones et al., 2018; Taschauer et al., 2020), the peptide with targeting and endosomal escape capacity, pH-/redox-responsive polymer for controlled release of RNAs, and so on. All applied biogenic vehicles and their cooperators serve the same goal: improving therapeutic efficacy and reducing off-target effects via enhancing tumor accumulation, immune escape and endosomal escape. In the following text, we will discuss their performance concerning these main aspects in detail. Of course, other special characteristics will also be described. We believe this paper will provide new and creative ideas to researchers involved in the fields of biogenic delivery and RNA-based therapies for cancer treatment.

### Oligonucleotides for RNA Delivery

Oligonucleotide assemblies, formed by DNA and RNA via non-covalent interactions within and between themselves, are nascent non-viral RNA carriers because of their unique programmable and predictable structure. Based on the high programmability of pairing combinations, oligonucleotides can form nanometer-scale structures with tunable physicochemical properties (e.g., size, shape, surface chemistry) to achieve optimal delivery effects by improving the tumor accumulation and cytosol release of RNAs (Kim et al., 2019). In addition, they can also be programmed with targeting ligands to produce tumor-specific delivery. All these characteristics as well as the intrinsic biocompatibility allow the well-designed oligonucleotide assemblies to exhibit negligible toxicity and immunogenicity (Hu et al., 2018). Most importantly, their inherent compatibility with therapeutic RNAs confers high capacity for RNA delivery via simple hybridization and prevents the need for positively charged transfection agents, which can induce severe side effects (Table 1).

**TABLE 1:** Oligonucleotides-based nano-sysetems for RNA delivery.

Vectors		RNA target	Specifics	References
DNA Assemblies	DNA	Bcl-2	None	Rahman et al.(2017)
	DNA	Survivin	None	Chen et al. (2015)
	DNA	VEGF	hairpin structure oligonucleotide for active targeting	Ren et al. (2016)
	DNA	PLK1	Aptamer AS1411 for active targeting	Lin et al. (2020)
	DNA	Braf	Aptamer AS1411 for active targeting	Xiao et al. (2021)
	DNA	PLK1	FA for active targeting; influenza hemagglutinin peptide for endosomal escape	Liu et al. (2021a)
	DNA	Bcl-2	DOX for combination therapy; TAT for cell-penetrating	Wang et al. (2021a)
		P-gp		
	DNA + Spermidine	mTOR	None	Wang et al. (2018a)
	DNA-PCL	PLK1	None	Ding et al. (2018)
	DNA-PCL	Hsp70	PDA for photothermal convertible agent	Ding et al. (2020a)
	DNA-PCL	EGFP	None	Xue et al. (2019)
RNA Assemblies	RNA + amino-modified CD	EpCAM	EpCAM aptamer for active targeting; sorafenib for combination therapy	Chen et al. (2020a)
	RNA	miR205, miR221	LXL-DNA aptamer for active targeting	Ding et al. (2020b)
	RNA + PEI	Bcl-2	DOX for combination therapy	Juneja et al. (2020)
	pRNA (3WJ)	MED1	HER2 aptamer for active trageting	Zhang et al. (2017)
	pRNA (3WJ)	XBP1	EGFR aptamer for active targeting	Zhang et al. (2021)
	pRNA (3WJ)	miR21	FA for active targeting	Lee et al. (2017)
	pRNA (3WJ)- cholesterol	miR21	FA for active targeting	Yin et al. (2019)
	pRNA (3WJ)- cholesterol	Survivin	PSMA and FA for active targeting, exosome for endosome escape	Pi et al. (2017)
	pRNA (3WJ)- cholesterol	Survivin	FA for active targeting, exosome for endosome escape	Zheng et al. (2019)
	pRNA (6WJ)	miR122	PTX for combination therapy, HTL for active targeting	Wang et al. (2021b)

Bcl-2: B-cell lymphoma-2; mTOR: mammalian target of rapamycin; VEGF: vascular endothelial growth factor; PLK1: polo-like kinase 1; P-gp: P-glycoprotein; Hsp70: heat-shock-protein 70; EGFP: enhanced green fluorescent protein; EpCAM: epithelial cell adhesion molecule; MED1: Mediator Subunit 1; XBP1: X-box-binding protein 1; HTL: hepatocyte targeting ligand; HER2: human epidermal growth factor receptor-2; EGFR: epidermal Growth Factor Receptor PSMA: prostate-specific membrane antigen.

### DNA Assemblies

DNA nanotechnology was proposed in the 1980s by Nadrian Seeman, but no construct was successfully built until 1998. Subsequently, various techniques have been developed to prepare DNA self-assemblies with assorted sizes, shapes, and structures (Li et al., 2021). Further studies confirmed that the internalization and gene silencing efficiency are heavily dependent on the size, shape, and structure of DNA nanostructures. To obtain a desirable Bcl-2 siRNA delivery system, Rahman et al. prepared eight DNA nanostructures (Rahman et al., 2017). The nanostructures with varied shapes and sizes exhibited differences in internalization efficiency, silencing effect, and anti-cancer activity. For example, smaller DNA nanostructures exhibited slightly higher internalization efficiency than the larger ones, and smaller rectangular structures displayed the highest gene silencing effect in both cell and animal models. Moreover, the shape of DNA nanostructures also affects their endosomal escape capacity. For example, the DNA nanoribbons were confirmed to be able to escape from endosomal entrapment, which may be attributable to their rigid structure and high aspect ratio, thereby permitting DNA nanoribbons to stretch out of endosomes (Chen et al., 2015). Thus, by tuning the shape and size using DNA nanotechnology, the internalization efficiency and endosomal escape capacity of RNAs can be significantly improved.

Although the internalization efficiency can be improved by tuning the characteristics of DNA nanosystems, most of them are endocytosed via non-specific pathways such as scavenger receptor- (Rahman et al., 2017), clathrin-, and lipid raft-mediated endocytosis (Chen et al., 2015), which can be executed by both cancer and normal cells. Thus, the systems with no precise targeting moieties can possibly trigger the release of various cytokines and immune stimulation (Rahman et al., 2017). To minimize off-target effects and improve the therapeutic effect by controlling RNA release in targeted cells, Ren and co-workers decorated an oligonucleotide nanovehicle with DNA primer hairpin for active targeting (Ren et al., 2016). By recognizing the receptors on the targeted cell surface, accurate RNA delivery and negligible off-target toxicity were guaranteed. As mentioned previously (Chen et al., 2015), the formed system with tube-like features also exhibits a high aspect ratio, which permits efficient endosomal escape to facilitate the release of RNA in the cytoplasm (Ren et al., 2016). In addition to nucleotide-based targeting agents such as the DNA primer hairpin mentioned above and the most widely applied AS1411 aptamer (Lin et al., 2020; Xiao et al., 2021), the chemical targeting ligand folate (Liu J. et al., 2021) and redox-sensitive disulfide bonds (Wang H. et al., 2021) have also been applied to improve the specific intracellular delivery and controlled release of RNAs. In these systems, peptides including influenza hemagglutinin peptide and transactivator of transcription peptide are added to improve the endosomal escape (Liu S. et al., 2021) and intratumoral delivery of RNAs (Wang Z. et al., 2021). The precise delivery, controlled release, and efficient endosomal escape of RNAs ensure effective cytotoxicity and tumor inhibition without observable systematic toxicity.

**FIGURE 3:**
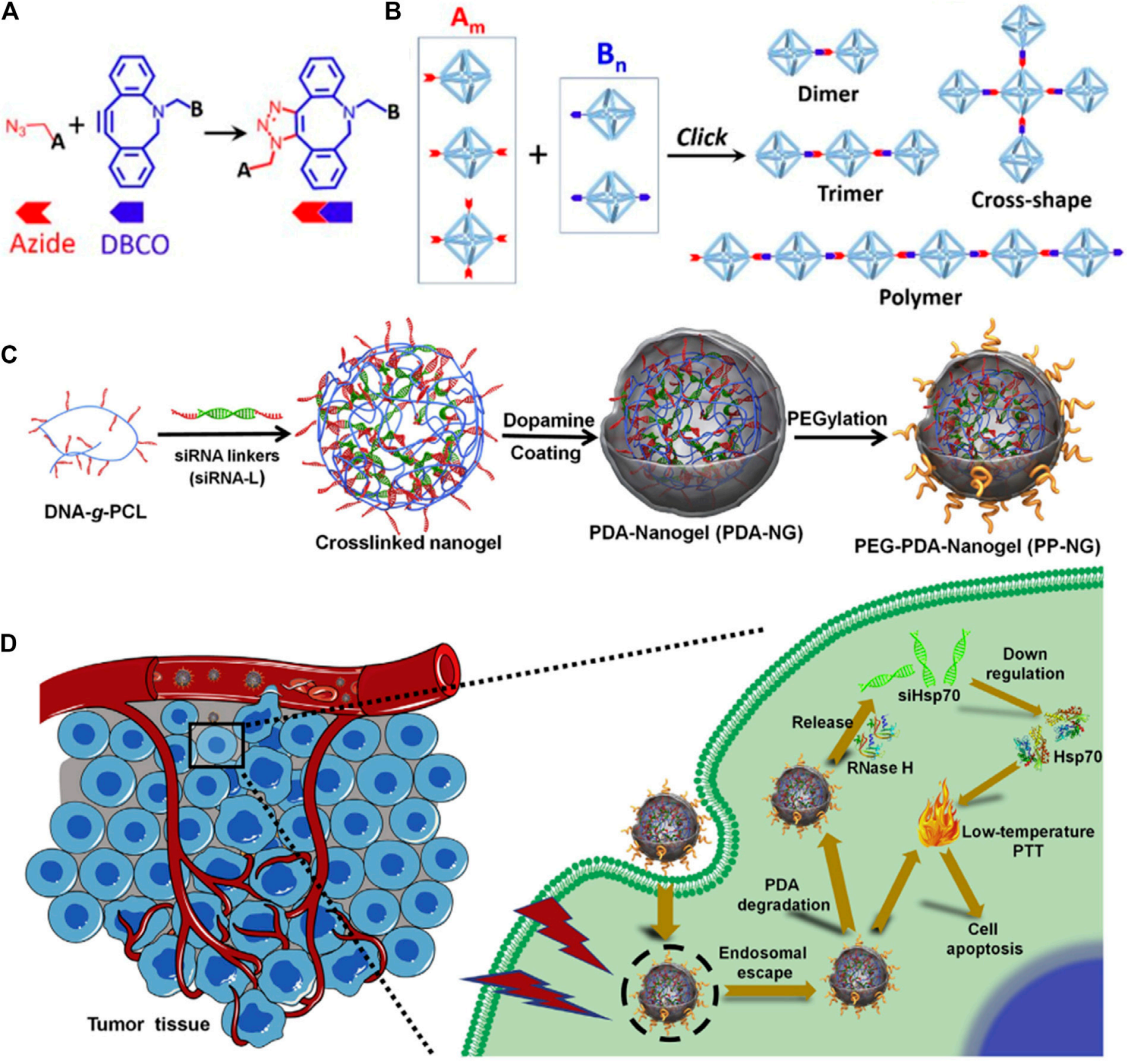
Preparation and application of chemically modify DNA strands for RNAs delivery. **(A)** Schematic illustration of copper-free azide-alkyne click reaction between azide and DBCO. **(B)** The DNA structures formed by click reaction with controlled architectures, such as dimer, trimer, cross-shape, and 1D polymer DNA frame (Lin et al., 2019). **(C)** Synthesis route of PDA-coated nucleic acid nanogel. **(D)** Schematic illustration of endosomal escape and PTT induced by PDA (Ding L. et al., 2020).

In addition to targeting, the applicability of DNA nanostructures is also limited by the labile “soft-matter” constructs in harsh physiological environments. Moreover, physiological environments that contain a high amount of DNase and low concentration of salt can induce the disintegrity of DNA nanostructures. To improve the stability, the spermidine/DNA nanoprism complex was developed (Wang D. et al., 2018). The results confirmed that this nanosystem shows high thermal stability and enzymatic resistance because of the addition of spermidine, which can serve as a protective layer for DNA. In addition, cationic spermidine can also shield the negative charge of DNA and thus improve its cellular uptake. Another strategy to improve stability is to chemically modify component DNA strands using copper-free click chemistry between azide and dibenzocyclooctyl (DBCO) (Figures 3A,B) (Lin et al., 2019), such as the modified DNA-grafted polycaprolactone (PCL) proposed by the Zhang group. In addition, the designed DNA-PCL with high stability can then be applied for siRNAs delivery via simple hybridization between DNA-g-PCL and siRNA linker (Ding et al., 2018; Xue et al., 2019; Ding F. et al., 2020). To increase the endosomal escape capacity, the photothermal convertible agent polydopamine (PDA) has been added which can also produce photothermal therapy (PTT) (Figures 3C,D) (Ding F. et al., 2020). After being entrapped by endosome, the delivery complex can be released into cytoplasm under laser radiation and further degraded to release Hsp70 siRNA. The combination of PTT and RNAi leads to an enhanced anti-tumor effect.

### RNA Assemblies

Compared with DNA nanotechnology, RNA nanotechnology emerged somewhat later in the 1990s (Høiberg et al., 2019). However, some advantages, especially their ability to be cloned and synthesized in large quantities, make them more feasible than DNA structures. The RNA nanostructures with compact structures have been confirmed to display much greater thermostability and serum stability than unfolded RNA strands (Qi et al., 2020), and thus, they can act as thermodynamically stable vectors of RNAs for intracellular delivery. In addition, they can also be programmed with therapeutic RNAs that can be transformed into active RNAs for RNAi through the cleavage of the Dicer enzyme. This particular characteristic makes the RNA nanostructures act as both vehicle and therapeutic biomolecule (Jang et al., 2018). And like DNA nanostructures, the RNA nanostructures can also be programmed with targeting ligands for specific delivery of RNAs.

**FIGURE 4:**
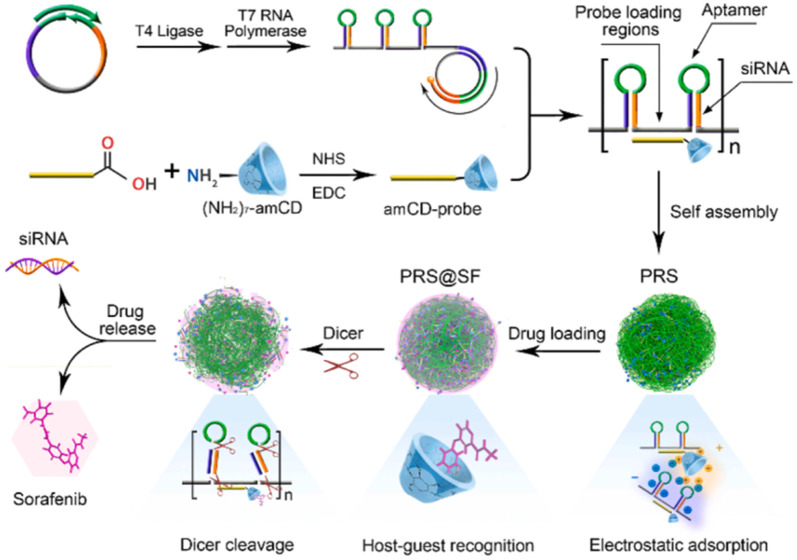
Schematic illustration of construction and Dicer-triggered disassembly of the SF-loaded porous RNA nanospheres (PRS@SF) (Chen HY. et al., 2020).

**FIGURE 5:**
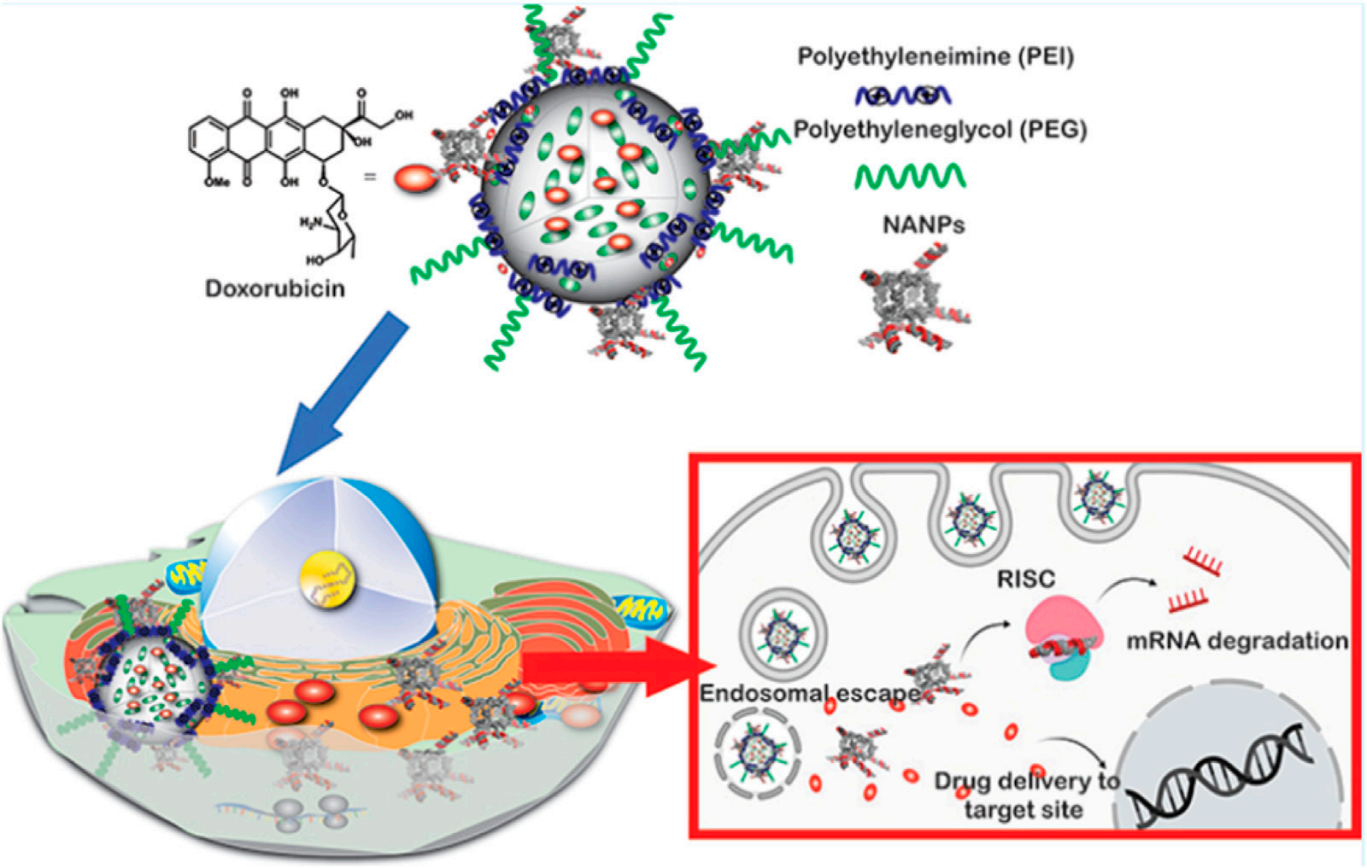
Schematic illustration of PEI-modified nucleic acid nanoparticles (NANPs) and its capacity in endosomal escape (Juneja et al., 2020).

For example, the porous RNA nanospheres (PRS) which is programmed with therapeutic siRNA targeting cell adhesion molecule (EpCAM) for drug resistance reversal and EpCAM aptamer for specific guidance was reported by Chen and co-workers. After entering targeted cells with the help of the aptamer, the PRS can be digested by Dicer enzymes in the cytoplasm, thus releasing EpCAM siRNA to induce a synergistic effect with sorafenib (SF) (Figure 4) (Chen HY. et al., 2020). Another similar system was also reported by Ding and co-workers for CXCR4 siRNA delivery, except that two miRNAs (miRNA-205 and miRNA-221) for tumor inhibition and a cholesterol-modified LXL-DNA aptamer for cancer cell targeting were also added through Watson-Crick pairing and Hoogsteen hydrogen bonding (Ding L. et al., 2020). It is confirmed that the hydrophobic cholesterol enhanced the stability of RNA nanostructures against nuclease attack and induced higher uptake efficiency. Absorbing the cationic polymer PEI-coated mesoporous silica nanoparticles (MSNP) onto RNA nanostructures *via* electrostatic interaction is also reported and confirmed to improve the stability of RNA assemblies (Juneja et al., 2020). The mechanism may be similar to the aforementioned system that the PEI-decorated layer can protect the RNA nanostructures from being degraded by nuclease (Wang D. et al., 2018). In addition, the PEI improve the endosomal escape capacity of the system owing to the “proton sponge” effect (Figure 5). In particular, the workers have also verified the influence of the shape of RNA nanostructures on their silencing efficacy and immunostimulatory activity, and the results demonstrated that globular structures exhibit the maximum immunostimulation, whereas fibrous structures display the greatest ability to suppress immunostimulatory effects and enhance gene-silencing efficacy (Juneja et al., 2020). This again verifies that the tunable RNA nanostructure displays a significant role in biological processes as shown in DNA nanostructure.

**FIGURE 6:**
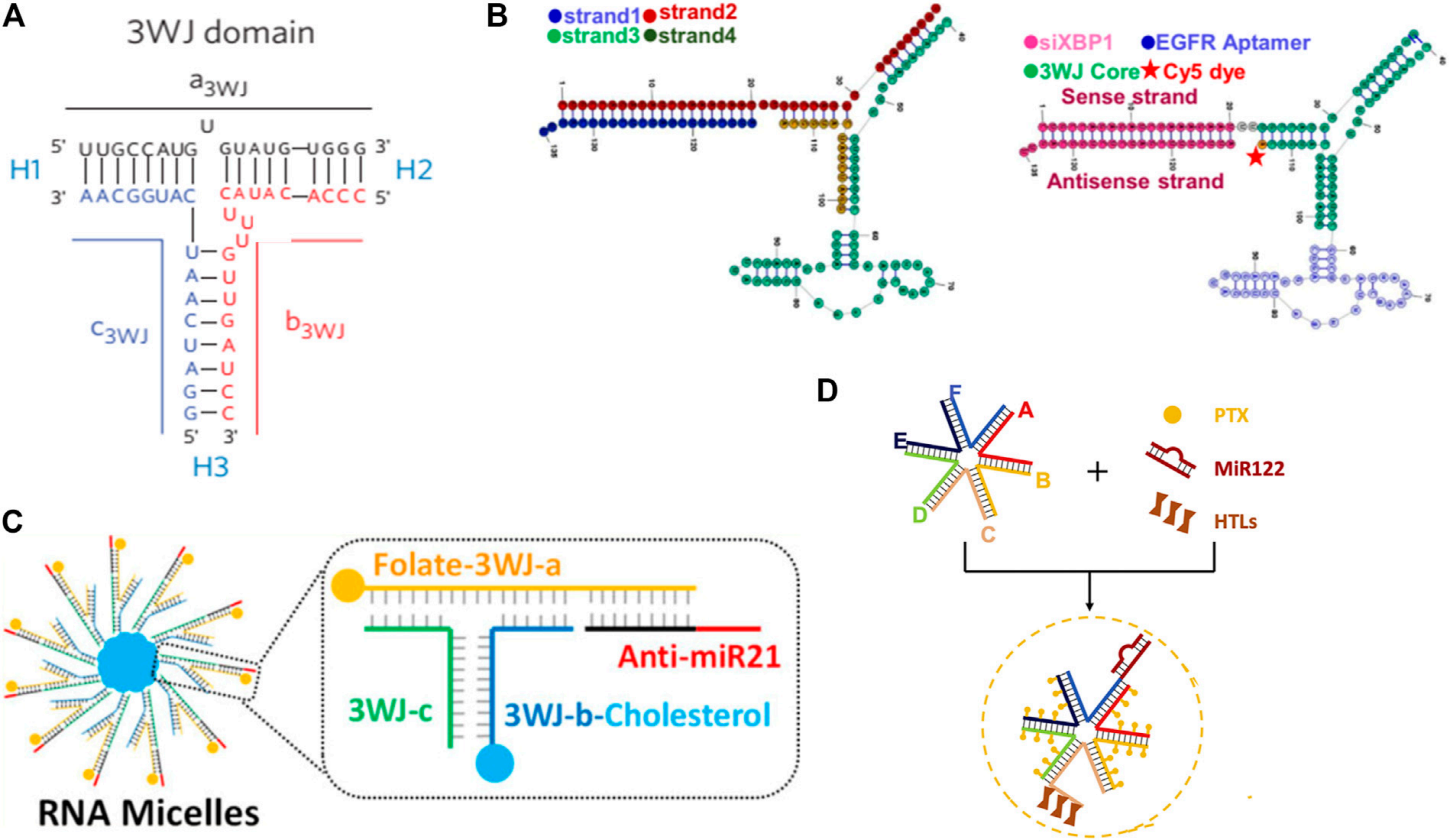
Illustration of RNA nanostructure formed by pRNA. **(A)** Struture of 3WJ domain composed of three RNA oligomers (Shu et al., 2011). **(B)** Scheme of EGFR-modified 3WJ-pRNA for siRNA targeted delivery (Zhang et al., 2021). **(C)** Structure of RNA micelles formed by cholesterol-decorated 3WJ-pRNA for miRNA delivery (Yin et al., 2019). **(D)** Newly developed 6WJ-pRNA for drugs and miRNA targeted delivery (Wang H. et al., 2021).

A special RNA nanostructure formed by packaging RNA (pRNA) from the phi29 DNA packaging motor system was widely engineered and applied for RNA targeted delivery by Guo group (Yoo et al., 2021). The pRNA is composed of two functional domains which are connected by a three-way junction (3WJ) region, and the 3WJ region of pRNA can be assembled from three pieces of small RNA oligomers with high affinity denoted as a_3WJ_, b_3WJ_, and c_3WJ_ (Shu et al., 2011). This unique structure confers high thermodynamical and chemical stability to pRNA, and they can keep integrality in the presence of 8 M urea or even *in vivo* after systemic delivery. In addition, because the 3WJ region is formed by two separate domains and replacing the helical domain with functional moieties does not affect the structure, folding, or intermolecular interactions of pRNA (Figure 6A) (Shu et al., 2011), many 3WJ-pRNA are modified with multiple functions for cancer treatment. For example, the aptamers human epidermal growth factor receptor-2 (Zhang et al., 2017), epidermal growth factor receptor (EGFR) (Figure 6B) (Zhang et al., 2021), and folate (Lee et al., 2017) have been conjugated to 3WJ-pRNA for the targeted delivery of RNAs to tumor tissues, and the specifically delivered RNAs significantly regressed tumor growth and improved survival. In addition to these targeting ligands, hydrophobic molecules such as chemotherapeutic paclitaxel (PTX) (Shu et al., 2018) and cholesterol (Figure 6C) (Yin et al., 2019) have also been conjugated to 3WJ-pRNA branches to make pRNA amphipathic, allowing it to assemble into micelles in water spontaneously. Both the systems exhibit enhanced anticancer effect and reduced side effects of RNAs. This may be not only because the EPR effect caused by the nanometer size and spherical shape, but also due to the rubber or amoeba-like deformation property of these RNA nanostructures. The special property enables them to squeeze out of the leaky vasculature to improve EPR effect while allowing the intact RNA nanoparticles to squeeze through renal filters for urine excretion resulting in no toxicity (Ghimire et al., 2020). To achieve endosomal escape capacity and release the therapeutic RNAs into the cytosol, 3WJ-pRNA has been combined with exosomes (Pi et al., 2017; Zheng et al., 2019). Exosomes not only facilitate the 3WJ-pRNA escape from the endosomal entrapment, but also improve the internalization efficacy of 3WJ-pRNA direct fusion with the cell membrane through tetraspanin domains (Pi et al., 2017). With further study, the Guo Lab reported a more stable 6-way junction (6WJ) scaffold using six component RNA strands (Figure 6D) (Wang Z. et al., 2021). The 6WJ scaffold enables the conjugation of multiple copies of drug molecules PTX along with various functional modules including miRNA 122 and hepatocyte targeting ligands (HTL) without affecting structural stability. The formed delivery system demonstrated high therapeutic efficacy and undetectable immunogenicity and toxicity. This was mainly attributable to the intrinsic properties of pRNA including high thermodynamic stability and chemical stability, which allows RNA structures to remain intact *in vivo* to avoid non-specific toxic drugs release, as well as the rubber-like deformation property to improve the EPR effect and renal filters for urine excretion, as demonstrated in their previous report (Ghimire et al., 2020).

### Exosomes for RNA Delivery

Exosomes, natural extracellular membrane vesicles consisting of hydrophilic cores and phospholipid bilayers, have been successfully used for RNAs delivery in cancer therapy because of their inherent delivery ability to transmit exogenous RNAs and suitable particle size ranging from 30 to 150 nm (Table 2). As mentioned above (Pi et al., 2017), they also display high capacity to escape from endosomal entrapment via membrane fusion with endosome. Exosomes can be secreted from different cell types, including tissue, blood, immune, and cancer cells. The origin of cells controls the properties of exosomes. For example, the cancer cell-derived exosomes appear to participate in tumor growth and metastasis, and regulate immune responses (Huang et al., 2019). Especially, the specific targeting capabilities (Nabariya et al., 2020) make them widely applied as delivery vehicles for cancer therapeutic agents. In addition, the RNAs loaded in cancer cell-derived exosomes can be transferred to the tumor site successfully due to the homologous tropism of exosomes (Xu et al., 2021). Compared with the cationic polymer PEI, exosomes display much lower organ toxicity than PEI due to their high biocompatibility. In addition to this, they also display faster and higher internalization than lipofectamine (Xu et al., 2021) and clinically approved state-of-the-art DLin-MC3-DMA LNP (Murphy et al., 2021). This may be because the existence of proteins on exosome membranes (e.g., tetraspanins and fusogenic proteins) which are reported to aid in cellular uptake. The other widely studied mesenchymal stem cell (MSC)-derived exosomes tend to exhibit immunomodulatory and tissue-regenerative properties, making them applicable as therapeutic tools for various diseases even including COVID-19 pneumonia (Muraca et al., 2020). Similar to cancer cell-derived exosomes, MSC-derived exosomes also display high tumor targeting ability (Abello et al., 2019; Guo et al., 2019). This may be due to the intimate interaction between MSCs and tumor tissues, and thus the targeted co-delivery of oxaliplatin (OXA) which can induce immunogenic cell death (ICD) and galectin-9 siRNA which is responsible for tumor immunosuppression reversal induces a significant synergistic effect for pancreatic ductal adenocarcinoma treatment (Zhou et al., 2021).

**TABLE 2:** Exosomes-based nano-systems for RNA delivery.

Cell source	Isolation method	Loading method	RNA target	Specifics	References
PANC-1 cells	Centrifugation	Electroporation	PAK4	None	Xu et al. (2020)
MSC	Centrifugation	Electroporation	Galectin-9	OXA prodrug for ICD-trigger	Zhou et al. (2021)
mesenchymal cells	Centrifugation	Electroporation	KRAS	None	Kamerkar et al. (2017)
HEK 293 T cells	Centrifugation or Isolation Kit	Electroporation	Cltc	None	Wan et al. (2020)
HEK 293 T cells	Centrifugation	Transfection	HGF	None	Zhang et al. (2018)
MSC	Centrifugation	Transfection	GRP78	Sorafenib for combination therapy	Li et al. (2018)
MSC	Centrifugation	Transfection	miR101	None	Zhang et al. (2020a)
bEND.3 cell	Isolation Kit	Transfection	VEGF	None	Yang et al. (2017)
HEK293 T cells	Centrifugation	Transfection	BCR-ABL	IL-3 for active trageting	Bellavia et al. (2017)
MDA-MB-231 cells	Isolation Kit	Transfection	miR126	None	Nie et al. (2020)
Milk	Centrifugation	Incubation	KRAS	FA for active targeting; PEI for condensing RNA	Munagala et al. (2021)
Neuro2A cells, DC	Centrifugation	Incubation	HuR	Cholesterol was conjugated to RNAs	O’Loughlin et al. (2017)
MCF-10 A cells	Serial extrusion	Electroporation	CDK4	None	Yang et al. (2016)
NIH3T3 cells	Serial extrusion	Electroporation	c-Myc	None	Lunavat et al. (2016)

PAK4: P21-activated kinase 4; Cltc: clathrin heavy chain; HGF: hepatocyte growth factor; GRP78: glucose regulated p rotein 78 kD; HuR: human antigen R; CDK, 4: cyclin-dependent kinases 4

**FIGURE 7:**
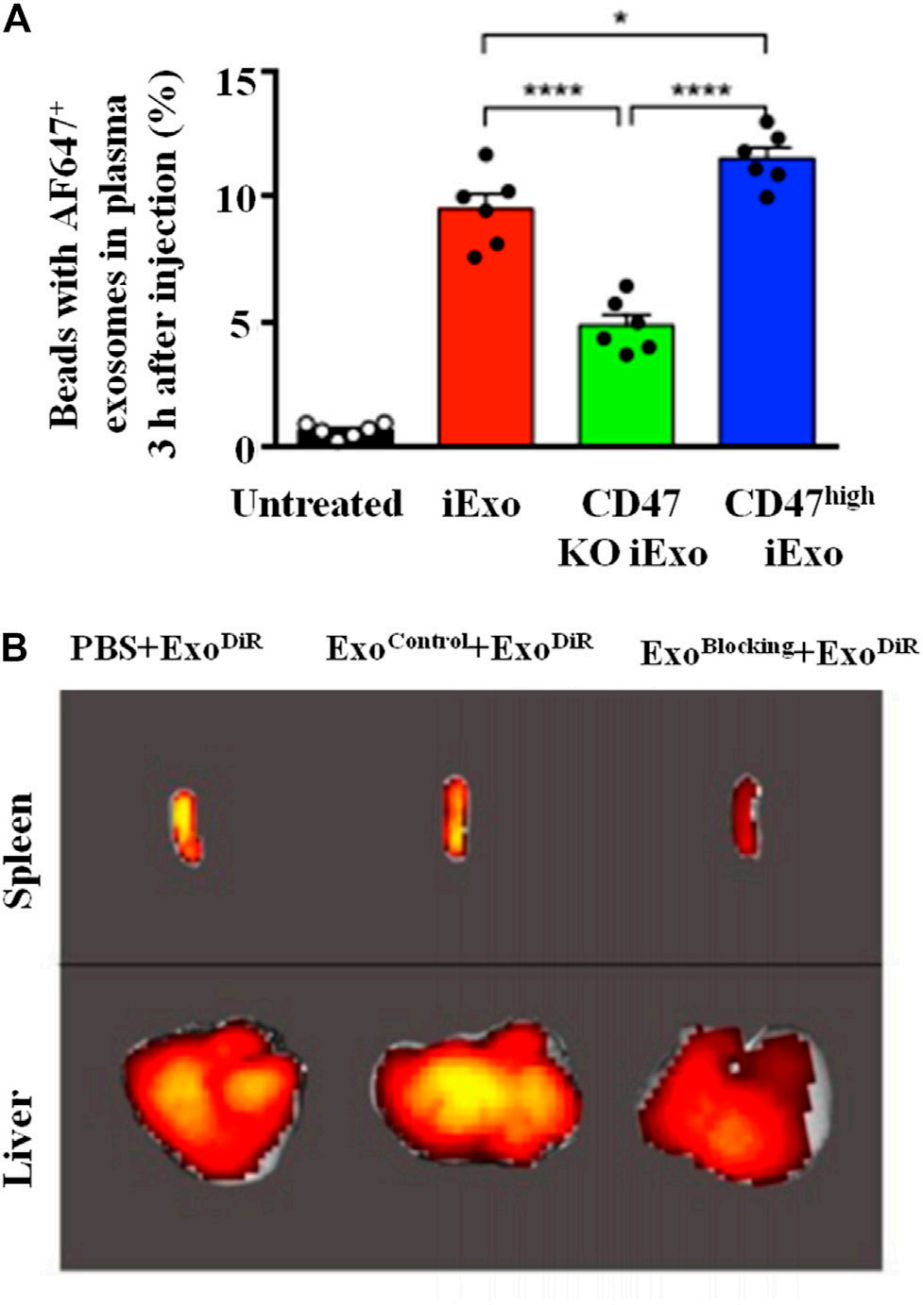
Mechanism of exosomes in immune escape. **(A)** CD47 protects exosomes from being cleared by monocytes and macrophages: flow cytometry analysis of AF647-tagged siRNA in exosomes with normal/knockout (KO)/high-expression CD47 in the circulation (Kamerkar et al., 2017). **(B)** Cltc blocking inhibits the macrophages endocytosis and decreases the accumulation of DiR-loaded exosomes in liver and spleen **(C)** (Wan et al., 2020).

Except the high ability in tumor targeting and internalization, exosomes also exhibit superior escape from phagocytic clearance compared to synthesized NPs. This is mainly related with the presence of exosomal protein CD47 which can initiate the “don’t eat me” signal to inhibit phagocytosis, and thus protect exosomes from being cleared by monocytes and macrophages (Figure 7) (Kamerkar et al., 2017). In addition, it has been pronounced that the high level of Cltc (clathrin heavy chain), which is closely related to the endocytosis of exosomes by macrophages, are expressed higher on macrophages than other endocytosis-associated genes. The pre-injection of Cltc siRNA-loaded exosomes can prevent the endocytosis of therapeutic exosomes loaded with miR-21a by macrophages significantly (Wan et al., 2020). In addition, the efficient escape from the immune system can decrease the accumulation of RNA-loaded exosomes in liver and spleen while increase that in the targeted site (Figure 6B), this leads to good biosafety and enhanced therapeutic effects.

**FIGURE 8:**
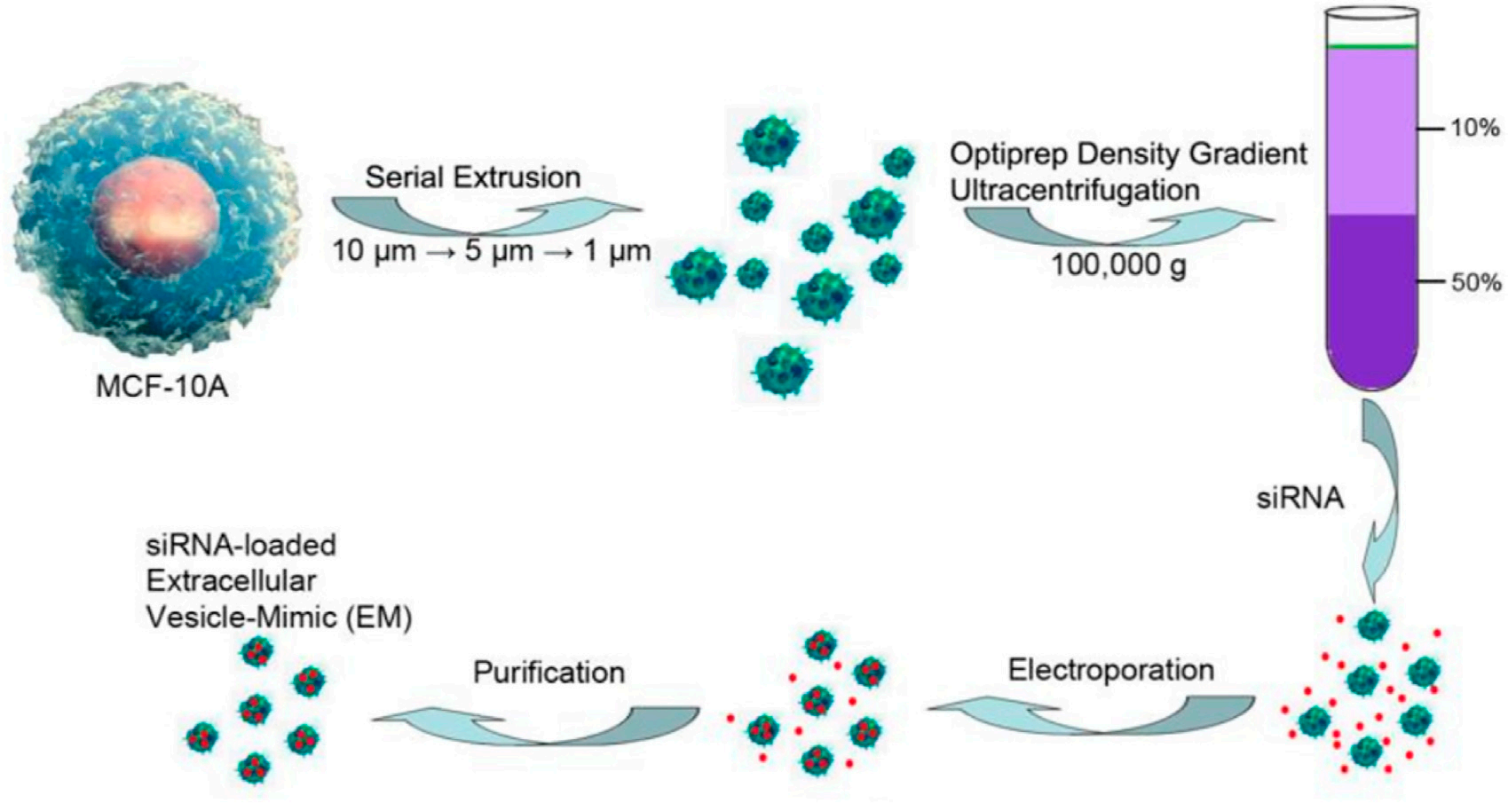
Preparation of exosome-mimics nanovesicles via serial extrusion and loaded with siRNA using electroporation method (Yang et al., 2016).

However, the biggest limitation for exosomes in wide application and clinical transformation is that there is no standard for effective isolation and purification which leads to a low production yield of exosomes. In addition, the fragility of RNAs and exosomes and the hydrophilcity of RNAs also result in low loading efficiency. Therefore, efforts have been devoted to improving the production yield and loading efficiency. To improve the production yield of exosomes, several methods have been developed, such as ultracentrifugation, co-precipitation, size-exclusion chromatography, and field-flow fractionation (Shao et al., 2018). Among these techniques, ultracentrifugation and co-precipitation appear to be two of the most widely used and simple approaches. The ultrahigh-speed centrifugation techniques can separate exosomes and other components based on their differences in density and size under different centrifugal forces, whereas the co-precipitation techniques achieve this using the hydrophilic polymers such as polyethylene glycol (PEG), which can decrease the solubility of exosomes. However, both methods have some disadvantages, such as time-consuming protocols, costly instrumentation, low recovery, and low purity for ultracentrifugation and difficulty in scaling and low purity for co-precipitation (Jiang et al., 2020). To realize the large-scale production, the exosome-mimics nanovesicles prepared by serial extrusion of cells has been reported (Figure 8) (Lunavat et al., 2016; Yang et al., 2016). The yield of these prepared exosome mimics is 150-fold higher than that of exosomes from the same cells, while maintaining similar physicochemical properties and anti-cancer effects compared with exosomes (Yang et al., 2016). But low purity and integrality of exosomes still exist in the process. Thus, methods for isolation and purification are still urgently needed, and new isolation technologies are being developed constantly.

The methods used for RNAs loading include incubation, ultrasonication, electroporation, transfection, and many other newly developed technologies. Electroporation and sonication are two of the most widely used methods based on physical mechanical force to load RNA into exosomes. The mechanical force induced under the electrical field or by sonicator probe can produce temporary pores on the membrane of the exosome, and the temporary pores facilitate the influx of RNA into the exosomes. It has been confirmed that electroporation does not disturb the stability of exosomal membrane structure and RNA and the disturbed membranes can be restored at 37 °C within an hour (Kim HJ. et al., 2016). In addition, since RNAs are relatively large and they cannot diffuse out the exosome spontaneously as small hydrophobic molecules do, the methods are particularly suitable for RNAs loading (Kamerkar et al., 2017; Wan et al., 2020; Xu et al., 2021; Zhou et al., 2021).

Different from the aforementioned methods using mechanical shear force, transfection is used to load RNAs into exosomes with the assistance of transfection agents. It induces lower mechanical damage to exosomes than that using mechanical force. Based on the formation process, the transfection method can be divided into pre-transfection and post-transfection. The former method describes the loading of RNAs during exosomes biogenesis (Li et al., 2018; Zhang et al., 2018; Zhang K. et al., 2020). Post-transfection refers to the loading of RNAs using transfection reagent (Bellavia et al., 2017; Yang et al., 2017; Nie et al., 2020) after exosome isolation. By comparation, the former approach is limited by cytotoxicity, poor quality, and inefficiency. First, the RNAs transfected into the donor cell may induce cytotoxicity and influence the production of exosome. Second, we cannot make sure that all the collected exosomes contain the transfected RNAs. More than that, the bioactivity of transfected RNAs can be destroyed in the donor cell. Thus, the post-transfection is more widely applied for RNAs delivery.

Among all methods for loading RNAs into exosomes, incubation technology appears to be the simplest strategy, requiring no special device or transfection agents. RNAs can fuse into the exosomes along the concentration gradient when they are incubated with exosomes without disturbing the exosomal membrane (Pham et al., 2021). However, this simple method exhibits lower loading efficiency than other methods. To overcome this obstacle, the physical addition of cationic PEI has been reported by Munagala and co-workers (2021). In addition, PEI improves the transfection efficiency of RNA several-fold compared with electroporation and transfection without changing the size and physical properties of exosomes. The chemical method involving the modification of RNAs with cholesterol to form hydrophobic RNAs has been widely studied, and hydrophobic RNAs can then insert into the lipid bilayer of exosomes via a mild incubation method (Didiot et al., 2016; Stremersch et al., 2016; O’Loughlin et al., 2017; Haraszti et al., 2018). Hydrophobic RNAs modification both enhances the loading efficiency of RNA and improves the stability of RNA (Didiot et al., 2016). It has also been verified that the cholesterol-modified RNA has no influence on physical and functional properties of exosomes.

### Cell Membranes for RNA Delivery

Like exosomal membranes, which carry a number of surface proteins to ensure accumulation in the target cell types, cell membranes also display intrinsic biological targeting ability and capacity to escape from the immune system. The source of the cell membranes determines its specific characteristics. For example, the most widely researched cell membranes are derived from cancer cells, which can be easily obtained through *in vitro* cell culture, have been proven to possess the capacity for immune escape and homing to tumor sites (Li et al., 2020; Shao et al., 2020), and erythrocytes, which are the most abundant cells with fewer organelles, were demonstrated to preserve long systematic circulation (Xie et al., 2019; Li et al., 2020). In addition, compared with the finding exosomes, cell membranes can be formed in a simpler, larger-scale manner with higher yield. Cell membranes are usually prepared by destroying the source cells, which are then centrifuged to remove the intracellular contents and collect the cell membranes. Finally, the obtained cell membranes are extruded through polycarbonate membranes to obtain the unified cell membrane vesicles, which are then combined with NPs via co-extrusion (Zhang L. et al., 2020; Xu et al., 2020), sonication (Zhao et al., 2020), or incubation (Wang et al., 2019) for multifunctional cancer treatment (Table 3). However, the low ability in endosomal escape seems to be one of the biggest limitations of cell membranes in RNAs delivery. In this part, we will discuss the specific characteristic of cell membranes derived from different types of cells, and their combination with other vectors for endosomal escape, fast internalization, and other functions.

**TABLE 3:** Cell membranes-based nano-systems for RNA delivery.

Cell source	Loading method	RNA target	RNA complex	Specifics	Ref
H1299 cells	Extrusion	PLK1	RNA/PBAE complex	PBAE with pH sensitivity	Zhang et al. (2020b)
HeLa cells	Extrusion	E7	RNA/PEI complex- loaded PLGA NPs	PTX for combination therapy	Xu et al. (2020)
HeLa cells	Sonication	Ca^2+^ channel	RNA/CS complex	DOX for combination therapy	Zhao et al. (2020)
4T1 cells	Extrusion	Bcl-2	RNA/PAMAM-CD	SN-38 for combination therapy	Fei et al. (2020)
RBC	Extrusion	Survivin	RNA/cationic BSA	RGD for active targeting	Wang et al. (2018b)
RBC	Extrusion	IL-1α	RNA/poly-L-histidine-grafted black phosphorus	PTX for combination therapy	Ou et al. (2019)
RBC	Extrusion	PLK1	RNA/citraconic anhydride grafted PLL	Angiopep 2 peptide for active targeting	Liu et al. (2020)
RBC	Extrusion	P-gp	RNA-cholesterol	DOX for combination therapy; aptamer AS1411 for active targeting	Wang et al. (2019)
Platelet	Extrusion	Survivin	RNA-loaded MOF	MOF with pH sensitivity	Zhuang et al. (2020)
ER	Ultrasound	EGFR	RNA/cationic lipid vesicle	None	Qiu et al. (2019)
Leukocyte	Sonication	LPCAT1	RNA/PEI/lipid vesicle	DOX for combination therapy	Jun et al. (2020)
PCa cells and BMSCs	Ultrasound	SREBP1	RNA/cross-linked peptide-lipoic acid micelle	DTX for combination therapy	Chen et al. (2020a)

IL-1α: Interleukin-1α; LPCAT1: lysophosphatidylcholine Acyltransferase 1; SREBP1: sterol regulatory element-binding protein 1

**FIGURE 9:**
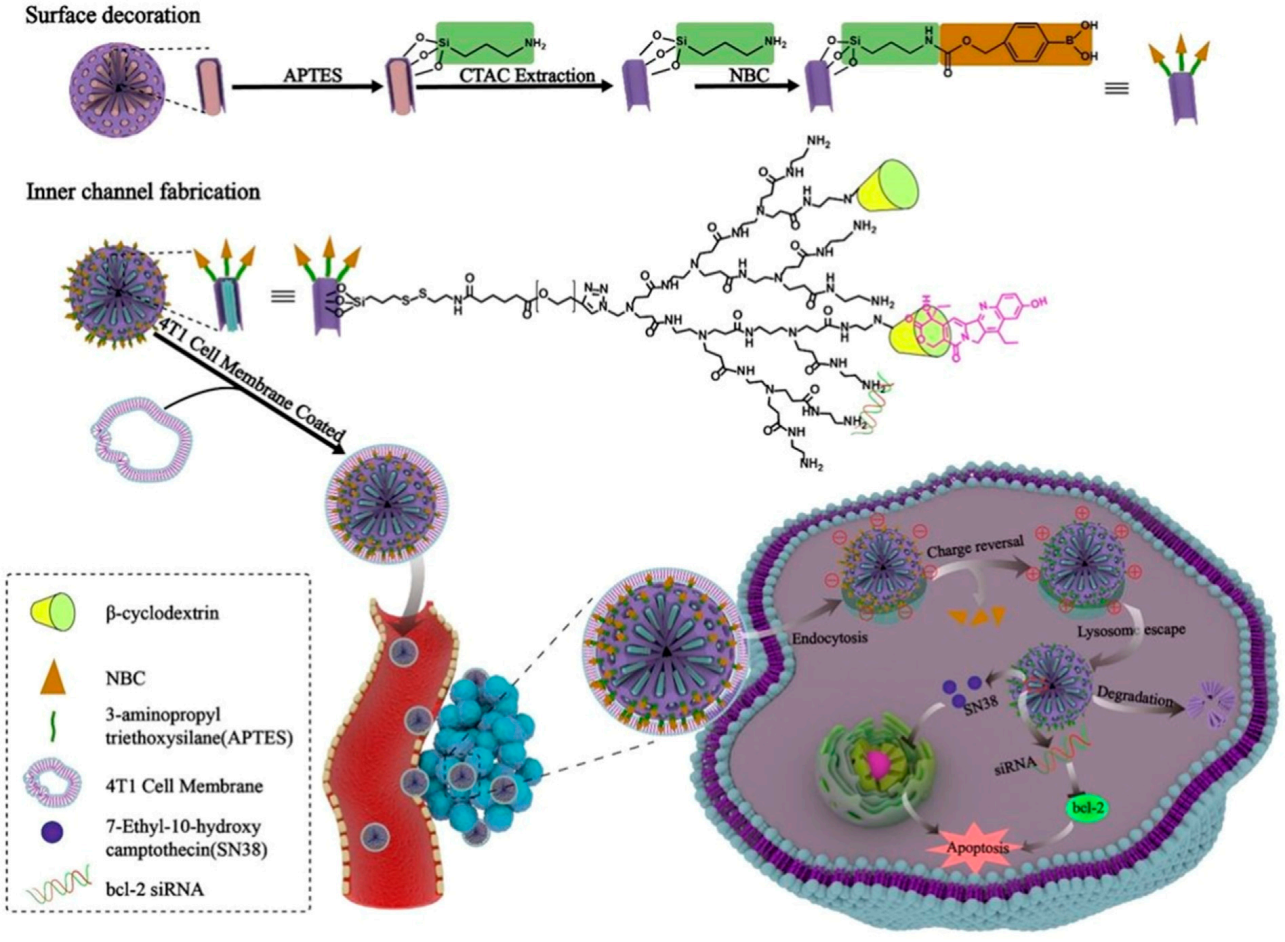
The SN-38 and siRNA delivery system decorated with CCM and ROS-sensitive NBC exhibit high capacity in tumor targeting and endosomal escape (Fei et al., 2020).

As for the application of cancer cell membranes (CCM) which exhibit good intrinsic targeting properties, the membranes of the human non-small cell lung carcinoma cell H1299 have been coated on siRNA/Poly (β-amino ester) (PBAE) core through a co-extrusion method (Zhang K. et al., 2020). The coating of CCM highly improves the stability of siRNA complexes in blood circulation, the escape from immune systems, and the specific targeting to homotypic cancer cells. In addition, accumulation in cancer cells, the synthesized PBAE can shift from hydrophobicity at neutral pH to hydrophilicity in an acidic environment, causing lysosome to burst and releasing siRNA into the cytosol. Both the targeting and endosomal escape capacities enhance the accumulation of siRNA in cancer sites for efficient gene silencing and tumor inhibition. In a special system, the cyclodextrin-PAMAM, which is applied for siRNA condensation and SN-38 loading, is conjugated with MSNP via disulfide linkers as the redox-liable inner core. The inner core is then modified and coated with ROS-sensitive nitrophenyl benzyl carbonate (NBC) moieties and 4T1 CCM. After internalization into 4T1 cells by the homotypic tropism of 4T1 CCM and located in lysosome, the ROS-sensitive negatively charged NBC moieties would be removed and the resultant electrostatic deshielding could facilitate their lysosomal escape by proton sponge effect (Figure 9). In addition, after entering into cytosol, the redox-liable disulfide linker is cleaved under the high concentration of glutathione to release the therapeutic cargos siRNA and SN-38 for synergistic therapy (Fei et al., 2020).

**FIGURE 10:**
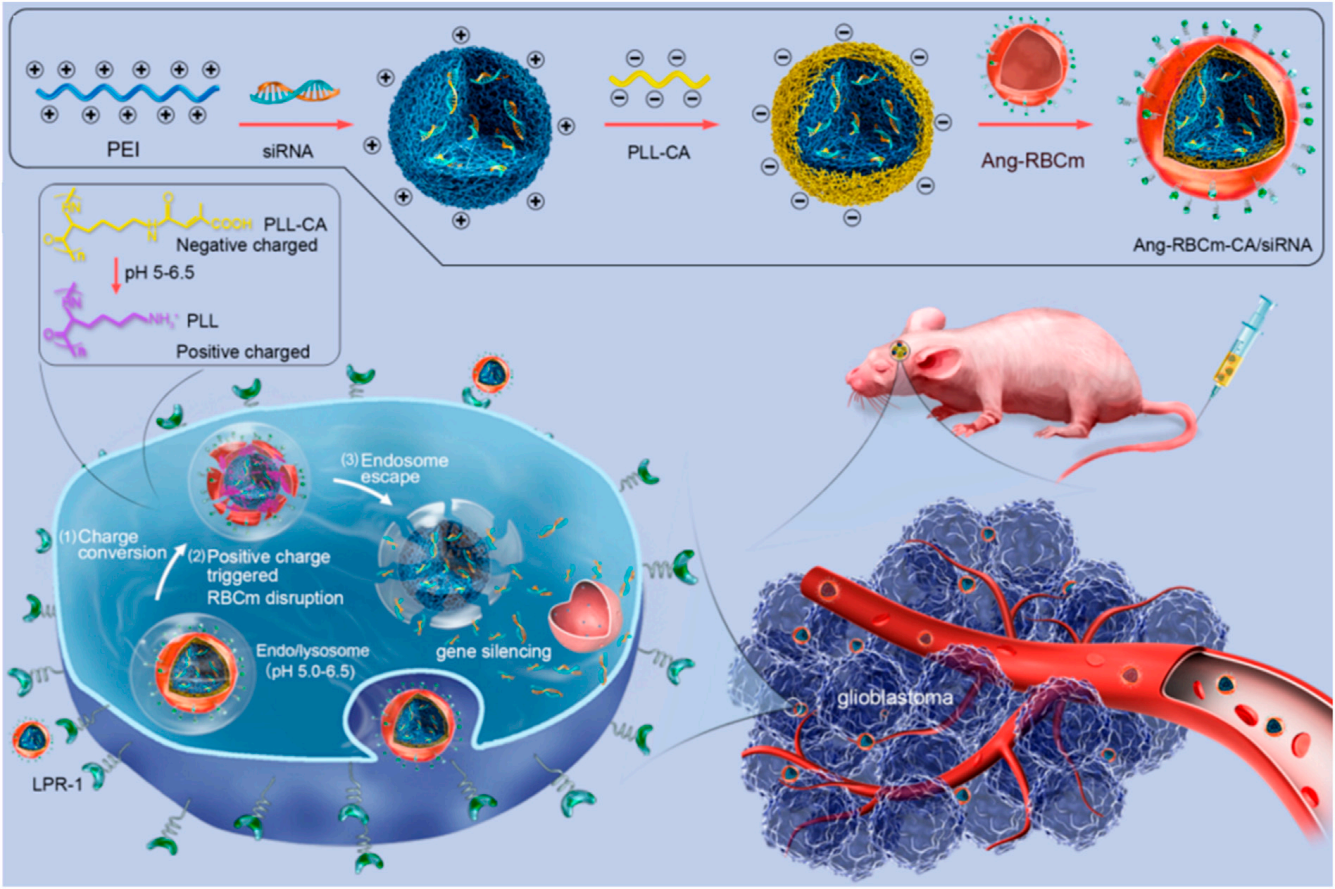
Schematic illustration of Angiopep 2 peptide-decorated charge-conversional biomimetic nanocomplexes with efficient endosomal escape for glioblastoma treatment (Liu et al., 2020).

As mentioned above, red blood cell membrane (RBCm) can provide long systematic circulation, and they have also been confirmed to display no toxicity or immunogenicity in autologous applications (Wang D. et al., 2018). But because lack of specific targeting ability, the RBCm are usually decorated with targeting ligand (Wang Y. et al., 2018; Ou et al., 2019; Wang et al., 2019; Liu et al., 2020). To reduce damage to membranes, the friendly method by incubating membranes with cholesterol-modified aptamer AS1411 has been reported by Wang and co-workers (2019). In addition, the therapeutic RNA is also loaded in the same manner. It has been confirmed that the cholesterol modification improves the aptamer and RNAs fixation with fast and high efficiency. Moreover, the efficient modification with AS1411 facilitates the cellular uptake and tumor specificity of RNA-loaded RBC membranes for enhanced tumor inhibition. To improve the ability to escape from the endosomal entrapment, the near-infrared (NIR)-activated poly (L-histidine)-grafted black phosphorus (Ou et al., 2019), as well as charge-reversible PEI-modified BSA (Wang Y. et al., 2018) and anhydride grafted poly-lysine (PLL-CA) (Liu et al., 2020) are applied. In the NIR-activated nanosystem, the ROS (mainly singlet oxygen) produced by the photosensitizer under light-activation can oxidize and rupture the membranes of exosomes, leading to the subsequent release of the therapeutics into cytosol. While the charge-reversible PEI-modified BSA and PLL-CA express negative charges at neutral pH value and turn positive at endosomal pH values, they induce the disruption of endosome to release RNAs into cytosol for gene silencing (Figure 10). In addition to improving endosomal escape capacity, the system formed by cationic BSA with worm-like shape seems to stay longer in the blood circulation and enhance accumulation of therapeutics within tumors than spherical ones. This may be due to the high aspect ratios and minimal regions of curvature the worm-like structure possesses.

**FIGURE 11:**
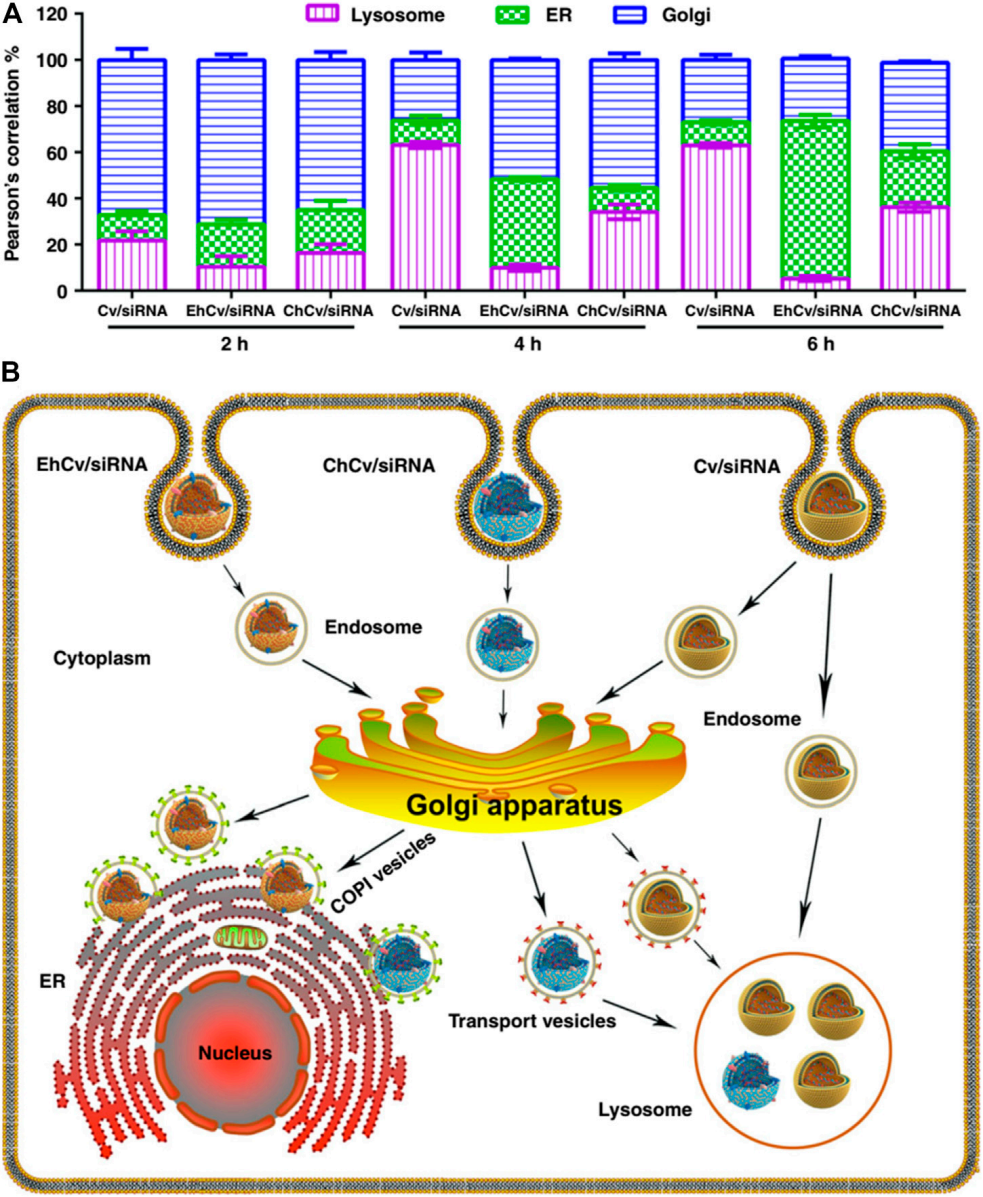
Intracellular trafficking pathways of siRNA-loaded NPs (Qiu et al., 2019). **(A)** The targeting ability of EhCv/siRNA NPs via endosome-Golgi-ER pathway and avoid the lysosome. **(B)** The intracellular trafficking pathways of different NPs.

Other cell membranes derived from platelets (Zhuang et al., 2020), endoplasmic reticulum (ER) (Qiu et al., 2019), and proinflammatory leukocytes (Jun et al., 2020) have also been researched for RNA delivery with specific targeting capacity. Among them, the platelet membrane has been cooperated with zeolitic imidazolate framework-8 (ZIF-8). ZIF-8, a metal-organic framework, exhibits minimal toxicity, high loading efficiency for RNAs, and good pH-sensitivity which can facilitate the endosomal disruption and release RNA into the cytosol (Zhuang et al., 2020). In particular, the ER membrane displays a different mechanism for endosomal escape. The results of the experiments show that siRNA-loaded lipoplexes (Cv/siRNA NPs) and CCM decorated Cv/siRNA (ChCv/siRNA) have significant lower ER retention and higher lysosomes accumulation behavior than ER coated Cv/siRNA (EhCv/siRNA) (Figure 11A). This says that EhCv/siRNA did transport siRNA through the endosome–Golgi–ER pathway (Figure 11B), and thus avoiding the lysosomal degradation to enhance the therapeutic efficiency of siRNA (Qiu et al., 2019). As illustrated by the aforementioned systems, membranes derived from different cells possess particular properties. To combine the different properties in a single system for multifunction activity, hybrid cell membranes have been developed (Chen J. et al., 2020). For example, membranes from homologous homing prostate cancer cells and bone marrow MSC have been fused and applied for bone metastatic castration-resistant prostate cancer treatment (Chen X. et al., 2020). It has been confirmed that the fused membrane provides bone-cancer dual targeting ability and high tumor permeability.

## Conclusion

Concerning RNAs therapy, it has been proposed and researched for decades. However, the intrinsic barriers in stability, immunogenicity, specific targeting, and endosomal escape make them not easy to realize clinical transformation. The approved RNA-drugs using nanoparticles shed new light on innovative design of therapeutics, but other vehicles are still needed to reduce immunogenicity while improving specific targeting and endosomal escape of RNAs. In this review, we summarized the RNA-delivery systems using biogenic materials for cancer treatment. These materials display excellent biocompatibility and biodegradability, resulting in low immunogenicity. In addition, their specifically intrinsic properties result in differences in therapeutic efficiency and many other biological functions among RNA delivery systems. For example, the oligonucleotides formed DNA and RNA NPs can deliver RNAs via simple Watson-Crick pairing and Hoogsteen hydrogen bonding with no need for additional positively charged transfection agents or complicated preparation technology. In addition, their unique programmable and predictable structure confers NPs with tunable size and shape, which can be optimized to induce high internalization efficiency as well as endosomal escape. In addition, they can also be programmed with functional sites for ligand attachments to ensure tumor-specific delivery. Concerning exosomes and cell membranes, they both exhibit homologous tropism for specific RNAs delivery. In addition, the biocompatibility with endosomal membranes also confers them with endosomal escape capacity via a fusion-based mechanism. In a word, all described biogenic materials can significantly reduce the immunogenicity and improve specific targeting and endosomal escape of RNAs.

Without question, there are some issues and challenges that need to be addressed and tackled before biogenic material-based systems are used for RNAs delivery in clinic. For example, it is important to better understand the *in vivo* behavior of RNA nanoparticles using different delivery vehicles. Especially, the relatively unstable oligonucleotides assemblies which lack covalent binding or crosslinking may dissociate at ultra-low concentrations in animal and human circulation systems after systemic injection. Although we can ensure their specific delivery to tumor sites *in vitro* or even *in vivo*, but for clinical applications, additional approaches for the selective uptake of RNAs by specific organs and diseased cells are desired. In addition, biogenic materials with high purity and large-scale production are basic requirements. However, none of these biogenic materials meets these requirements, especially exosomes. In addition, the contents of exosomes such as nucleic acids and proteins participate in both physiological and pathological processes. Their ambiguous roles make them so they cannot be applied for RNA delivery for sure. For example, the most contentious MSC-derived exosomes, many reports have confirmed their therapeutic effect for cancer treatment, but some studies demonstrate their significant roles in the growth, progression, and metastasis of tumors. Therefore, it is important to clarify the biological function of these materials before their clinical use. In summary, all biogenic materials have their strengths and weaknesses, and additional efforts are required to develop ideal carriers for RNA delivery, especially carriers with clinical applicability.
